# Comparison of 5-year outcomes and quality of life between endovenous laser (980 nm) and microwave ablation combined with high ligation for varicose veins

**DOI:** 10.3389/fsurg.2022.1022439

**Published:** 2022-10-21

**Authors:** Pengcheng Fan, Longlong Cong, Jian Dong, Yang Han, Lin Yang

**Affiliations:** Department of Vascular Surgery, the First Affiliated Hospital of Xi’an Jiaotong University, Xi’an, China

**Keywords:** varicose veins, laser ablation, microwave ablation, long-term outcome varicose veins, long-term outcome, high ligation

## Abstract

Our study aims to evaluateand compare the long-term results of endovenous laser (EVLA) and microwave ablation (EMA) combined with high ligation in treating varicose veins (VVs). A total of 122 patients (150 legs) underwent EMA combined with high ligation, and 127 patients (167 legs) underwent EVLA procedures (980 nm) combined with high ligation in this retrospective study. Outcomes included the Aberdeen Varicose Vein Questionnaire (AVVQ) score, the Venous Clinical Severity Score (VCSS), clinical recurrence of VVs and patient satisfaction duringthe 5-year follow-up.During the 5-year follow-up, patients who underwent the EVLA procedure showed a higher recurrence of VVs than those who underwent the EMA procedure (22.75% vs. 13.33%, *P *= 0.03, odds ratio (OR): 1.91, 95% confidence interval (CI): 1.06–3.45), especially at the primary site (6% vs. 14.37%, *P *= 0.01; OR: 2.63; 95% CI: 1.21–5.72). VV recurrence within 3 years was higher in patients who underwent EVLA than in those who underwent the EMA procedure (73.68% vs. 40%, *P *= 0.01; OR: 4.2; 95% CI: 1.37–12.86). Compared with those at baseline, the AVVQ score, VCSS and EQ-5D score improved significantly at 5 years for patients who underwent either procedure (*P *< 0.01); however, the VCSS and AVVQ score were higher for patients who underwent the EVLA procedure (*P *= 0.05). The patient reintervention rate was higher for EVLA than for EMA (14.79% vs. 7.33%, *P *= 0.033; OR: 2.19; 95% CI: 2.06–5.34). Our results confirmed that EMA and EVLA improve the QoL of patients and that EMA combined with high ligation demonstrates lower 5-year recurrence, especially at primary sites.

## Introduction

Endovenous thermal ablation techniques offer faster postoperative recovery with enhanced quality of life (QoL) than traditional high ligation and stripping procedures for varicose veins (VVs) (1–4). Thus, the thermal ablation procedure has become the first-line therapy recommended by the guidelines for VVs and is widely used worldwide (5, 6). Previously published literature has revealed that endovenous microwave ablation (EMA) was a safe and efficacious endovenous thermal procedure for the treatment of VVs and resulted in fewer complications and lower recurrence at 12 months than endovenous laser ablation (EVLA) (7, 8).

However, no data on the comparative long-term efficiency between EMA and EVLA have been published thus far. In this study, the aim was to evaluate the 5-year follow-up results of EMA vs. EVLA in terms of the Aberdeen Varicose Vein Questionnaire (AVVQ) score, the venous clinical severity score (VCSS) and the recurrence of VVs.

## Material and methods

### Patients

From January 2014 to December 2015, 249 patients (317 legs) with VVs were included in a retrospective study from university-affiliated hospitals; the EMA group included 122 patients (150 legs), and the EVLA group included 127 patients (167 legs). All patients were diagnosed according to clinical manifestations and venous ultrasonography (Philips-HD7, EIN, NL), and all VVs and perforator veins were marked before surgery. The study complied with the Declaration of Helsinki for investigation in humans and was approved by the Ethical Committee of The First Affiliated Hospital of Xi'an Jiaotong University, and all patients provided written informed consent.

The inclusion criteria were primary symptomatic VVs with great saphenous vein (GSV) incompetence under a duplex ultrasound scan (reflux time of GSV >0.5 s) and a VV diameter less than 12 mm (saphenous hiatus or knee medial). The exclusion criteria were duplication of the GSV or an incompetent anterior accessory saphenous vein, small saphenous vein or deep vein insufficiency, deep vein thrombosis history, arterial stenosis or occlusion, and refusal to undergo the endovenous procedure.

### Treatment procedures

The same team of doctors completed all procedures, and the EVLA and EMA procedures were performed under tumescent local anesthesia. The GSV trunk and tributaries were ligated in the groin viaa conventional procedure, after which the endovenous ablation procedures were performed. Thetortuous varicesbelow the knee were treated by pin phlebectomyand sclerotherapyfor all patients.

### Endovenous ablation

Endovenous ablation was performed according to previous reports (3, 7, 8). A laser fiber or microwave wire was inserted to 2 cm below the saphenofemoral junction using the Seldinger technique in the GSV of the medial knee and then withdrawn during the ablation under tumescent anesthesia. In the EVLA group, thermal ablation was performed with a 980-nm laser using 12-watt power and a bare fiber with the goal of delivering 70 joules/cm to the vein. The GSV below the knee were intermittent ablated *via* laser ablation, perforators and obvious varices (diameter greater than 5 mm) were ablated *via* laser ablation (power 10 W) under tumescent local anesthesia. In the EMA group, the GSV procedure was performed similarly to the EVLA procedure using 50-watt power, and the treatment parameters were set according to previous reports (8, 9), the energydelivery to the VVs was estimated at around 80 joules/cm. Additionally, the perforators and obvious varices(diameter great than 4 mm) were ablated using a short microwave needle (power 20 W) or laser ablation.

After thermal ablation, the patient's legs were wrapped with a self-adhesive compression bandage for 48 h (10). Then, all patients were required to wear gradient compression stockings (25 mmHg, ankle, only day wear) for at least one month.

### Follow-up and outcome measures

Patients were invited to participate in a 5-year follow-up. Patients who had not participated in the outpatient follow-up were required to complete the questionnaire through an online video clinic visit and then complete the examinations at a local hospital. The clinical records ofall patients were recorded and analyzed bythe chief doctor of the research team, and all patients were evaluated by physicians during follow-up.

GSV recanalization of the great saphenous vein was defined as a length of the open segment of the ablated vein segment greater than 10 cm during the follow-up period. The closure failure or recurrence of perforator veins was defined as the previous report (11). All patients were followed-up at one month and every year after the procedure, and ultrasound examination was performed to measure the outcomes of the ablation procedures.

The primary outcomes measured at 5 years were patient-reported, disease-specific QoL and clinical recurrence. The effect on QoL was evaluated by the AVVQ (12, 13) and the EuroQol Group 5-Dimension Self-Report Questionnaire (EQ-5D) (14). The AVVQscore ranges from 0 to 100, where lower scores indicate betterQoL. The EQ-5D has five dimensions, whose scores range from −0.594 to 1.000; higher scores indicate betterQoL. Clinical recurrence was determined by the patient's symptoms,objective clinical andphysical examinations and ultrasound scans.The occurrence of any new varices at the primary ablation site or phlebectomies sites that had not recorded before the procedure were confirmed to have recurred (11, 15) and was defined as primary site recurrence, while the occurrence of new onset varicesat non-ablation sites or phlebectomies sites was defined as new onset recurrence. The patients were evaluated by an independent team of physicians (including doctors and nurses). The patients with clinical recurrence of varicose and obvious symptoms are recommended to be undergoing the re-intervention procedure (phlebectomy, foam sclerotherapy or ablation), and the additional treatment are selected according to the patient's wishes.

Secondary outcomes were clinical symptoms as assessed with the Venous Clinical Severity Score (Chinese version; score ranges from 0 to 30; 0 represents no significant venous disease, and 30 is the maximum score) (16). An additional outcome—patient 5-year satisfaction rate—was recorded for recurrence during follow-up, we provide three answers: Satisfied, Fair, and Non-satisfied and patients choose the answer based on their own wishes.

### Statistical analysis

All clinical parameters were obtained during preprocedure hospitalization and at follow-up visits and were collected in Excel software (Version 2010, Microsoft, Redmond, Washington). When all patients had completed the 5-year follow-up, the study analyses were performed *via* SPSS software (SPSS 12.0, Chicago, IL, United States). Categorical data form both procedures were compared *via* the chi-square test, numerical are were described as the mean (standard deviation), and hypothesis significance testing was performed with two-sample *t* tests (two-tailed). Clinical recurrence was analyzed by using Kaplan–Meier survival analysis, and the odds ratio and 95% confidence interval were calculated. *P *< 0.05 was described statistically significant.

## Results

### Patients and treatment

A total of 147 and 166 patients underwent the microwave and laser ablation procedures, respectively (technical success rate 100%, the closure rate of GSV was 100% at one month), of whom 122 (150 legs, 83%) and 127 (167 legs, 77%), respectively, completed the follow-up. Patient data were collected from the case notes.The baseline and clinical data are listed in Table 1; no significant differences were identified between the two procedures in terms of patient characteristics, comorbidities or clinical, etiological, anatomical, and pathophysiological (CEAP) classification (*P *> 0.05). Both legs were treated in 22.95% and 31.50% of the microwave and laser ablation procedures, respectively (*P *> 0.05). The average number of phlebectomy were similar in both procedure (EMA: 1.87 ± 0.97 vs. EVLA: 2.04 ± 0.96; *P *> 0.05).

**Table 1:** Baseline data of the study participants in both procedures.

	EMA (*n* = 122)	EVLA (*n* = 127)	*P* value*
Gender (M)	72 (59.02)	67 (52.76)	0.32
Age (years)	52.22 ± 8.62	51.35 ± 11.29	0.61
Weight (kg)	69.69 ± 12.17	67.12 ± 10.79	0.19
Limb location			
Right	38 (31.15)	31 (24.41)	0.26
Bilateral	28 (22.95)	40 (31.50)	0.13
CEAP			0.19^#^
C3	39	51	
C4	74	70	
C5	22	26	
C6	15	20	
Diameter of GSV (mm)	8.11 ± 2.35	7.86 ± 3.02	0.41^#^
Deep vein reflux no. (%)	23 (15.33)	22 (13.17)	0.58^#^
No. of perforators			
>2 perforators/limb	77	81	0.61
≤2 perforators/limb	73	86	
Location of perforators			0.47
Below the knee	123	142	
Above the knee	27	25	
Stroke	2	2	
Hypertension	14	20	
Diabetes type 2	5	6	
Chronic kidney disease	4	1	
Coronary atherosclerotic disease	2	1	
Other	0	2	

EMA, endovenous microwave ablation; EVLA, endovenous laser ablation; M, male; kg, kilogram; CEAP, clinical, etiological, anatomical, and pathophysiological; GSV, great saphenous vein; mm, millimeter; *N*, number.

* *P* value, EVLA compared with EMA.

^#^ *P* value, compared with no. of legs.

### Primary outcome

**Figure 1:**
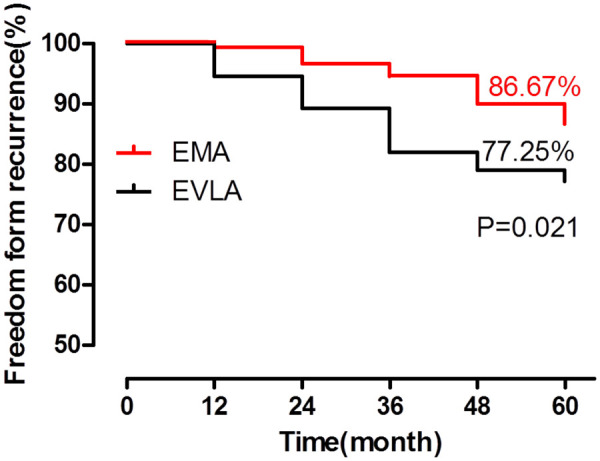
The EMA procedure resulted in fewer recurrent VVs than the EVLA procedure at 5 years and showed a higher freedom from recurrence (EMA: 86.67% vs. EVLA: 77.25%, *P *= 0.021, Kaplan–Meier analysis). *P < 0.05, compared with EVLA.

The results of the primary recurrence outcome indicators for both procedures during the 5-year follow-up are shown in Table 2. A high GSV closure rate was similarly observed between the EMA and EVLA procedures (98% vs. 96.41%, *P *> 0.05), and the recanalization rate of the GSV was similar (2% vs. 3.59%). Patients who underwent the EVLA procedure showed a higher 5-year VV recurrence rate than those who underwent the EMA procedure (22.75% vs. 13.33%, *P *= 0.03; odds ratio (OR): 1.91; 95% confidence interval (CI): 1.06–3.45), especially at the primary site (6% vs. 14.37%, *P *= 0.01; OR: 2.63; 95% CI: 1.21–5.72); thus, EMA showed a higher freedom from recurrence (Figure 1). New onset recurrence (OR: 1.16; 95% CI: 0.15–9.11) was similar between the patients treated with the EMA and EVLA procedures, and most recurrence sites were located below the knee (90% vs. 89.48%, *P *> 0.05). Interestingly, recurrence within 3 years was higher following EVLA than EMA (73.68% vs. 40%, *P* = 0.01; OR: 4.2; 95% CI: 1.37–12.86).

**Table 2:** The primary outcomes of the patients in both procedures.

	EMA (*n* = 150)	EVLA (*n* = 167)	*P* value*
Location of cannulation			0.56
Medial malleolus	113 (75.33)	121 (72.46)	
Below the knee	37 (24.67)	46 (27.54)	
Closure rate of GSV	147 (98.00)	161 (96.41)	0.61
Type of recurrence	20 (13.33)	38 (22.75)	0.03
Primary-site	9 (6.00)	24 (14.37)	0.01
New onset	11 (7.33)	14 (8.38)	0.89
Location of recurrence			0.48
Above the knee	0 (0)	2 (5.26)	
Popliteal	2 (10.00)	2 (5.26)	
Below the knee	18 (90.00)	34 (89.48)	
Time of recurrence			0.01
≤ 3 years	8 (40.00)	28 (73.68)	
3–5 years	12 (60.00)	10 (26.32)	

EMA, endovenous microwave ablation; EVLA, endovenous laser ablation; GSV, great saphenous vein; *N*, number.

* *P* value, EVLA compared with EMA.

**Figure 2:**
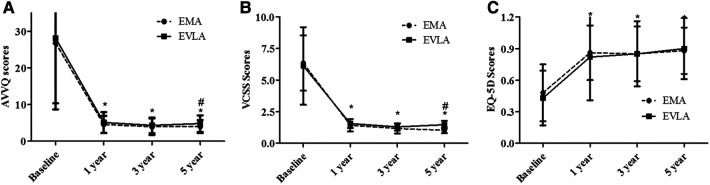
The QoL was measured by the Aberdeen varicose vein questionnaire (AVVQ) (**A**) and the euroQol group 5-dimension self-report questionnaire (EQ-5D) (**B**) scores for patients treated with the EMA or EVLA procedure. Compared with the baseline QoL scores, the postprocedure scores were improved following both procedures (*P *< 0.01). The Venous Clinical Severity Score (VCSS) (**C**) was lower postprocedure than at baseline (*P *< 0.01), and the AVVQ score and VCSS were higher in patients treated with EVLA than with EMA at 5 years. *P < 0.01, compared with baseline; ^#^*P *< 0.05, compared with EVLA.

The QoL outcome was evaluated by the AVVQ and EQ-5D scores (Figures 2A,B). Figure 2 summarizes the results for both primary QoL outcomes. Both procedures showed improvement in the two scores from the baseline scores (*P *< 0.01). There was no significant difference in the EQ-5Dscore between the patients in the two procedure groups (*P *> 0.05); however, the AVVQ scores were higher in patients who underwent EVLA than in those who underwent EMA (*P *= 0.05).

### Secondary outcomes

The post-procedure VCSS scores at 5 years of both procedures was significantly better than the corresponding baseline value (Figure 2C), while the post-procedure scores were lower than the corresponding baseline values, and the post-procedure VCSS scores were higher in EVLA patients than in EMA patients (*P *= 0.05). At 5 years, 25 legs treated with the EVLA procedure and 11 legs treated withthe EMA procedure (65.79% vs. 55%) underwent additional treatment in recurrent patients (Table 3). Overall, the reintervention rate of all patients was higher with EVLA than with EMA (14.79% vs. 7.33%, *P *= 0.033; OR: 2.19; 95% CI: 2.06–5.34). Regarding patient satisfaction, the patients who underwent the EMA procedure showed higher satisfaction (91.8% vs. 83.46%, *P *= 0.046; OR: 0.45; 95% CI: 0.21–0.98).

**Table 3:** The secondary outcomes of the patients in both procedures.

	EMA (*n* = 122)	EVLA (*n* = 127)	*P* value*
Reintervention rate	11 (7.33)	25 (14.79)	0.03^#^
Additional therapy	11	25	
Foam sclerotherapy	7	15	
Phlebectomy	3	8	
Thermal ablation	1	2	

EMA, endovenous microwave ablation; EVLA, endovenous laser ablation; *N*, number.

* *P* Value, EVLA compared with EMA.

^#^ *P* Value, compared with no. of legs.

## Discussion

EVLA is the first-line endovenous procedure recommended by the guidelines for the treatment of VV patients (11, 17); a previous report, however, revealed that EMA is a valid thermal ablation procedure for VVs. Compared with high ligation/stripping and EVLA, the EMA procedure exhibited a shorter operating time and fewer complications (7, 8). Our retrospective study demonstrates favorable long-term results for both the EMA and EVLA procedures, with significant improvements in clinical status and QoL during the 5-year follow-up. Moreover, our results indicate that EMA and EVLA show differences in the rates and sites of recurrence at 5 years; the recurrence rate at primary sites was higher with the EVLA procedure, while the new onset recurrence rate was similar. Furthermore, both procedures resulted in relatively high freedom from clinical recurrence—as indicated by the sable AVVQ and EQ-5D postprocedure scores—and higher patient satisfaction, even in patients who experienced recurrence and required additional therapy. What is more, in our clinical practice, EVLA or EMA procedure could be performed for each patient, and the final treatment option depends on the patient's willingness, because according to the Chinese medical law, the patients choose the therapy procedure according to their own willingness and the doctor's detailed explanation of all therapy procedures.

Our study revealed the higher GSV closure rates between the EMA and EVLA procedures, our results were consistent with previously reported for endovenous ablation procedures (18, 19). A recent report indicated that the clinical recurrence of VVs was higher in patients treated with the EVLA (980 nm wavelength) procedure without high ligation. These data illustrate that high ligation may be necessary to ensure the long-term effect of conventional thermal ablation procedures (20), therefore, we believe that high ligation could be helpful for most patients underwent the bare lasers or microwave ablation, especially in patients with larger diameter of GSV. Furthermore, our study demonstrates that the low recurrence rate of EMA mainly manifests within 3 years. The recurrence rate at the primary site from EVLA was higher than that from the EMA procedure, which may be related to the energy and power of the different thermal devices used for the procedures. Our results are consistent with a previous report, in which the author confirmed that primary site recurrence was more common after the EVLA procedure at 5 years (21). Other reports have confirmed that different energies and powers may also affect the long-term outcomes of the procedures (19, 22, 23). In addition, the different thermal mechanisms of EMA and EVLA may result in different clinical outcomes, and clinical recurrence may be caused by technical failure, strategic failure, neovascularization or disease progression (22–25). Recurrence after the ablation procedure might affect the long-term outcome, and clinical recurrence might require further therapy. Although both procedures showed high satisfaction rates, a lower reintervention rate was achieved with the EMA procedure in our study. A previous report showed the lowest recurrence rates following the RFA procedure, while the EVLA procedure showed the highest recurrence rates (26). Moreover, most recurrent VVs occurred below the knee in our study; thus, we believe that treating these VVs is very important. Additionally, the advantage of EMA in treating VVs below the knee has been reported in other studies (7, 8). Moreover, due to anatomical factors such as vein tortuosity, stab phlebectomy or sclerotherapy is also the effective adjuvant therapy when using endovenous ablation procedure, which is helpful to improve the clinical outcomes, previous report have also confirmed the feasibility and effectiveness of this technique (27).

VVs have a significant effect on QoL and limit daily activities and functional performance. Surgical and thermal ablation procedures can improve the QoL of patients after therapy, and most studies demonstrate no difference in QoL after different thermal procedures in most patients (28, 29). The QoL measure obtained following intervention for VVs can not only evaluate the improvement of individual patients but also be used to compare differences between different therapy modalities. In this study, our results confirmed that the AVVQ score and VCSS were better than the corresponding baseline values and that the EQ-5D scores were improved after 5 years for both the EMA and EVLA procedures. Both procedures demonstrated similar outcomes regarding patient QoL. These data demonstrate that both procedures are effective with regard to the long-term outcome and patient QoL; however, EVLA patients showed a higher recurrence rate and AVVQ score and VCSS than EMA patients.

### Limitations

Our study is a retrospective study of the 5-year results in the treatment of VVs between EMA and EVLA; nevertheless, it still has some shortcomings. The first limitation is the fact that it was not randomized or blinded. The second limitation is that the study is not a prospective study, and the conclusion may be affected by some selection bias. Moreover, this study mainly evaluated the recurrence rate and QoL in patients treated with EMA or EVLA; however, a relatively high number of patients did not attend all follow-up visits despite a scheduled visit and a reminder message.

## Conclusion

In conclusion, our study demonstrates that the EMA and EVLA combined with high ligation procedures improve patient QoL, and a lower recurrence rate was observed for patients in the EMA procedure group in a 5-year follow-up period, especially at the primary site. The higher frequency of recurrence after EVLA (980 nm) needs to be confirmed in future studies.

## Data Availability

The original contributions presented in the study are included in the article/Supplementary Material, further inquiries can be directed to the corresponding author/s.
